# Depressive and socially anxious symptoms, psychosocial maturity, and risk perception: Associations with risk-taking behaviour

**DOI:** 10.1371/journal.pone.0202423

**Published:** 2018-08-15

**Authors:** Adam N. Pailing, Renate L. E. P. Reniers

**Affiliations:** 1 College of Medical and Dental Sciences, University of Birmingham, Birmingham, United Kingdom; 2 Institute of Clinical Sciences, University of Birmingham, Birmingham, United Kingdom; 3 Institute for Mental Health, University of Birmingham, Birmingham, United Kingdom; Technion Israel Institute of Technology, ISRAEL

## Abstract

Risk-taking behaviour and onset of mental illness peak in adolescence and young adulthood. This study evaluated the interconnectedness of the domains of risk-taking behaviour, mental health (symptoms of depression and social anxiety), psychosocial maturity, risk perception, age, and gender in a sample of 306 adolescents and young adults. Participants between the ages of 16 and 35 completed online self-report measures assessing risk-taking behaviour, depressive symptoms, socially anxious symptoms, psychosocial maturity and risk perception. Socially anxious symptoms, psychosocial maturity, and risk perception were directly associated with risk-taking behaviour. Correlations between depressive symptoms, socially anxious symptoms, and psychosocial maturity were found. Psychosocial maturity proved a better predictor of risk-taking behaviour than age in this cohort. The findings indicate that mental health impacts upon risk-taking behaviour and that consideration should be given to psychosocial maturity in attempts to reduce adolescent and young adult risk-taking behaviour.

## Introduction

Risk-taking includes behaviours that have a chance of a desired, beneficial outcome but with the possibility of unwanted, negative consequences [1,2]. Adolescence and young adulthood are characterised by a disproportionate increase in risk-taking behaviour [3–6] which only declines in adulthood [7–12]. Unintentional injuries, often caused by excessive risk-taking behaviour, are the leading cause of death in adolescents and young adults [13]. A greater understanding of factors influencing these behaviours is therefore essential and may aid in the prevention of accidents and mortalities.

Socioemotional factors such as the presence of peers [3,14–16], personality [17,18], and gender [15,19,20] have received ample attention in recent years, whilst factors such as mental health and psychosocial maturity have mostly stayed under the radar, despite their stated importance. In England in 2016/2017, an estimated 1 in 6 people experienced mental health problems, with 421,000 men and women between the ages of 18 to 35 receiving psychological treatment for a common mental disorder, such as depression or anxiety [21]. Adolescents and young adults experience a surge in mental illness unequalled by any other developmental stage, such that 83% of all mental illnesses commence before the age of 21 [22]. Studies have found that individuals at these ages who display depressive symptoms engage in health-related risk-taking such as drunk driving to a greater degree than individuals with good mental health [23–27]. Despite these findings showing a positive association between depressive symptoms and risk-taking behaviour, laboratory based studies utilising risk-taking tasks have produced contradicting findings, with depressive symptomatology being associated with reduced risk-taking behaviour [28,29]. Social anxiety almost exclusively first manifests in individuals younger than 25, with onset generally in late childhood or early adolescence [30]. Like depression, social anxiety is associated with risk averse behaviour in experimental tasks [17,31–34], though a growing body of evidence suggests a subset of socially anxious individuals develop risk prone behaviours to increase acceptance from others [35–38].

Differences in one’s developmental status or competence may present as social and emotional immaturity [39]. Risk-taking behaviour in adolescence and young adulthood is commonly associated with differences in maturational processes of socioemotional and cognitive control systems [17,40–42]. A developmental gap created by a rapid increase in affective reactivity and sensitivity to reward, contrasting with a much slower but steadier development of one’s abilities to self-regulate, may contribute to behaviour that can be labelled both risky and immature [43]. Yet, rather than placing the focus on psychosocial maturity as an approximate measure of development [39,44], a hallmark of research is the use of age differences to explain variation in risk-taking behaviour. As individuals develop at different rates, no one 17-year-old can be compared to another on the grounds of being developmentally equal and caution needs to be exercised if age is to be used as a proxy marker for development. The limited research using psychosocial maturity as an approximate measure of development has demonstrated that psychosocial maturity is better than age at predicting antisocial and delinquent behaviour in adolescents and juvenile offenders [44–46]. Research comparing psychosocial maturity and age as predictors of risk-taking behaviour is thus warranted.

Besides their association with risk-taking behaviour, an association between mental health and psychosocial maturity has demonstrated importance. The prevalence of depression reduces with maturity [47]. Likewise, symptoms of social phobia seem to improve with increased levels of maturity [48]. Conversely, depression has been linked to poorer impulse control [49], and social anxiety is associated with reduced aggressive behaviours [50], both aspects of psychosocial maturity. Psychosocial maturity could therefore, albeit speculatively, be a potential protective factor for mental health, warranting further investigation.

Risk perception is independent of an individual’s opportunity to take risks, instead it measures an individual’s preference towards risk-taking and is therefore inversely correlated with health-related risk-taking behaviour [17,51]. Mental health is well known to impact decision-making [52,53], and being emotionally immature may impact one’s decision to take a risk [40]. Because of its association with all these domains, perception of risk should be included in an investigation of domains influencing risk-taking behaviour.

The current study used path analysis to evaluate the interconnectedness of the domains of risk-taking behaviour, mental health (symptoms of depression and social anxiety), psychosocial maturity, risk perception, age, and gender in a sample of adolescents and young adults. As depressed individuals generally take fewer risks in tasks and questionnaires [28,29], depressive symptoms were predicted to be associated with decreased risk-taking behaviour. Risk perception tends to be reduced in individuals with depressive symptoms [51], therefore depressive symptoms were predicted to be associated with a decrease in risk perception. Socially anxious individuals are mainly risk avoidant [17,31–34], so socially anxious symptoms were predicted to be associated with decreased risk-taking behaviour and increased risk perception. Depressive and socially anxious symptoms were also predicted to be associated with each other, given their high co-prevalence [54,55]. As increased psychosocial maturity is associated with reduced antisocial and delinquent behaviour [44–46] and more mature decision-making [44], an association was predicted with reduced risk-taking behaviour and increased perception of risk. Concurrent with previous findings [17,51], increased risk perception was predicted to be associated with reduced risk-taking behaviour. As males tend to take more risks than females [17,20], this variable was additionally added to the model and associations were specified postulating males to display increased risk-taking behaviour and reduced risk perception compared to their female counterparts. To investigate whether psychosocial maturity is indeed a better predictor of risk-taking behaviour than age in this study, both domains were specified in the model. Research has displayed ageing to be associated with a reduction in risk-taking behaviour [7–12] and an increase in risk perception [9,12,56], so these associations were specified in the model.

## Methods

### Participants

The current study aimed to evaluate the interconnectedness of the domains as discussed above in a sample of adolescents and young adults. While adolescence commences at age 10 [57] recruitment of participants at this young age was not feasible due to logistical reasons (requirement of parent/legal guardian consent in an online study). Young people over 16 are presumed to be capable of giving consent on their own behalf, provided they have capacity to understand the specific circumstances and details of the research being proposed; this is highly dependent on the information presented to them. As detailed information about the study was presented online and contact details for the researchers were provided in case of any further queries, no consent from parents or legal guardians was required for participants aged 16 years and older. Young adulthood continues until a person is in their thirties [58] and therefore, the age range of participants recruited in the current study was 16–35 years old.

308 participants completed the online survey between the dates of 31^st^ January and 20^th^ March 2017. Participants were recruited using online (28.9%; *n* = 89), email (13.6%; *n* = 42), and poster (6.5%; *n* = 20) advertisement in Birmingham, Manchester, Nottingham, and Liverpool; and via the University of Birmingham Research Participation Scheme (19.8%; *n* = 61). Word of mouth recruited 27.3% (*n* = 84) of participants, and 3.9% (*n* = 12) were recruited through “other” means. Online advertisement included advertisement on social media and Gumtree. Posters were placed in university buildings and community locations such as libraries and community centres. All participants were given the opportunity to enter a prize draw to win one of five £20 Amazon vouchers. University of Birmingham undergraduate Psychology students received degree credits for their participation. Ethical approval was granted by the University of Birmingham Internal Ethics Review Committee (reference number Y16_C2_15_SJDL). This work was supported with a grant by the University of Birmingham Population Sciences and Humanities degree Programme.

Exclusion criteria included living outside of the United Kingdom. Two participants were excluded: one lived outside of the United Kingdom, and another was younger than 16. The remaining 306 participants (201 female; 103 male; 2 N/A) ranged between 16.01 and 35.85 years old (median age 20.46 years), of which 82.3% (*n* = 252) were White, 7.5% Asian (*n* = 23), 2.9% (*n* = 9) Black, 5.2% (*n* = 16) mixed, and 1.9% (*n* = 6) indicated “other”. The highest educational achievement was A-levels or equivalent for 63.7% (*n* = 195) of the sample, undergraduate degree for 18.6% (*n* = 57), postgraduate degree for 5.6% (*n* = 17), GCSEs for 3.9% (*n* = 12), Higher National Degree for 2.6% (*n* = 8), 2.9% (*n* = 9) selected other, and 2.6% (*n* = 8) had no school certificates.

### Procedure

All recruitment methods included a link to the survey which took potential participants to a page with information about the study. After potential participants had the chance to read and consider the information presented to them (with no time limit), informed consent for participation was sought. Participants who gave written informed consent were presented with demographic questions concerning gender, age, ethnicity, education, and how they heard about the study, followed by questionnaires and a task (described below). At the end of the survey, participants were directed to a page containing contact details of mental health support organisations. The entire procedure took on average between 20 and 30 minutes.

### Measures

#### Symptoms of depression

The Patient Reported Outcome Measurement Information System–Depression [59] has 8-items which ask participants to rank how regularly they have experienced a certain feeling (eg. “I felt helpless”) over the previous seven days on a 5-point Likert scale (1 = *Never*; 5 = *Always*). The measure demonstrated excellent internal reliability in previous studies (Cronbach’s alpha (α) = .95) [59,60] and this study (α = .95).

#### Symptoms of social anxiety

The Social Interaction Anxiety Scale (SIAS) and Social Phobia Scale (SPS) [61] measure social anxiety symptoms when holding interactions with individuals in social situations (eg. “I am nervous mixing with people I don’t know well”), and completing tasks in the presence of others (eg. “I can feel conspicuous standing in a queue”), respectively. Both scales have 20-items and are ranked on a 5-point Likert scale asking participants how characteristic each statement is of them (0 = *Not at all*; 4 = *Extremely*). The scales demonstrated excellent internal reliability in previous research (SIAS α = .92, SPS α = .94) [17] and in this study (SIAS α = .95, SPS α = .95). Sum scores for each scale were combined to give one total score representative of social anxiety.

#### Psychosocial maturity

Psychosocial maturity was measured using the model developed by Steinberg and Cauffman [39], which defines maturity as having three characteristics: temperance (the ability to assess a scenario, thereby controlling one’s impulses), perspective (being able to recognise a complex situation and make a decision within a wider context), and responsibility (developed with reliability and autonomy). Each of these characteristics has two measurable components (described below), leaving six scales which are used. An overall score is created by taking the average of the *z*-scores for temperance, perspective, and responsibility, and then creating a normalised scale from 0–5 from this average [44].

Temperance is measured using two subscales of the Weinberger Adjustment Inventory (WAI) [62]: Impulse Control, an 8-item subscale (eg. “I stop and think things through before I act”); and Suppression of Aggression, a 7-item subscale (eg. “I pick on people I don’t like”). Responses are ranked on a 5-point Likert scale (1 = *False or Mostly False*; 5 = *True or Mostly True*). Both subscales demonstrated adequate internal reliability in previous research (Impulse Control α = .76; Suppression of Aggression α = .78) [45] and the current study (Impulse Control α = .85; Suppression of Aggression α = .83). Scores from the Suppression of Aggression subscale are reversed and the total from the two scales are combined to obtain the temperance score.

Perspective is measured using the Consideration of Others subscale of the WAI [62] and the Consideration of Future Consequences Scale [63]. The Consideration of Others subscale is a 7-item scale used to measure the extent to which individuals account for others’ perspectives (eg. “I often go out of my way to do things for other people”). This subscale is measured in the same way as the other WAI subscales, and has demonstrated good internal reliability in previous research (α = .73) [44] and this study (α = .76). The Consideration of Future Consequences Scale is a 12-item scale which measures the ability of an individual to recognise future consequences (eg. “My convenience is a big factor in the decisions I make or the actions I take”). Participants rank on a 5-point Likert scale (1 = *Extremely uncharacteristic*; 5 = *Extremely characteristic*) how characteristic a behaviour is of them, with good reliability in previous research (α = .76) [63] and the current study (α = .86). The sum scores for each scale are standardised and then an average is taken to obtain the perspective score.

Responsibility is measured using the Personal Responsibility Scale, a subscale of the Psychosocial Maturity Inventory [64], and the Resistance to Peer Influence Scale [65]. The Personal Responsibility Scale asks participants their degree of agreement with 30 statements (eg. “If something more interesting comes along, I will usually stop any work I’m doing”) on a 4-point Likert scale (1 = *Strongly Disagree*; 4 = *Strongly Agree*). The scale is reverse scored, and then an overall score is calculated using the average of the items. The scale demonstrated good internal reliability in previous work (α = .83) [44] and in this study (α = .87).

The Resistance to Peer Influence Scale is a 10-item scale where participants are given 10 pairs of opposing statements (eg. “Some people take more risks when they are with their friends than they do when they are alone.” and “Other people act just as risky when they are alone as when they are with their friends”). Participants are asked to determine which statement is more characteristic of them, and then select whether that statement is *“Sort of True for Me”* or *“Really True for Me”*. Each item is scored between 1 and 4, with lower scores corresponding to reduced resistance to peer influence. Items are averaged to give an overall score, demonstrating good reliability in previous work (α = .73) [66] and in this study (α = .70). To obtain the responsibility value, the scores from each scale are standardised and an average taken.

#### Risk perception

An adapted version of the Benthin Risk Perception Scale was used in this study [67]. In this version, participants are given six risky behaviours and asked four different questions about each, corresponding to four subscales: Risk Appraisal (*Please indicate the likelihood of risk for this behaviour*), Seriousness of Consequences (*Please indicate how serious the consequences of this behaviour are if something bad happened as a result of it*), Affect (*Please indicate how scary you find this behaviour*), and Benefits vs Risks (*Please indicate whether the benefits outweigh the risks for this behaviour*). The behaviours included: smoking cigarettes, getting into a fight with another person, riding in a car with a drunk driver, vandalism, having sex without a condom, and shoplifting. “Drinking alcohol” is normally a behaviour measured in the scale. In this version it was replaced with “getting into a fight” as it is illegal in the United Kingdom for individuals under the age of 18 to drink alcohol, therefore they would be expected to assign considerably more risk to this scenario than someone older than 18. Responses are ranked on a 4-point Likert scale, with higher scores indicating a greater perception of risk. The average of all of the responses gives the risk perception value. The “getting into a fight” item has been successfully used in trials investigating similar populations to the one in this study (α ranging between .84 - .86) [68,69] and good internal consistency was found in this study too (α = .78).

#### Risk-taking behaviour

The study used two measures, the Bomb Risk Elicitation Task (BRET) and the RT-18. Scores from the BRET and RT-18 were converted into *z*-scores and an average of the two was taken to produce a composite risk-taking behaviour score. By combining a behavioural task and a self-reported measure, a more accurate representation of overall risk-taking behaviour could be assessed.

The BRET [70] asks participants to decide on a number of boxes they would like to collect out of 100 boxes. For each collected box, the participant gets one point. One of the 100 boxes contains a bomb; the location of this bomb is randomly determined and unknown, but it is equally likely to be in any of the 100 boxes. The boxes are numbered 1 through 100. Collection of boxes starts at number 1 and continues until the box whose number is equal to the number the participant chose. For example, a participant who chooses number 13 collects boxes 1 to 13. If the number of the box in which the bomb is located is equal to or smaller than the number chosen (for example, the participant chooses number 13 and the bomb is located in box 6), the bomb is collected resulting in zero earnings. If the bomb is located in a box with a number higher than that chosen by the participant (for example, the participant chooses number 13 and the bomb is in box 68), the participant earns one point for each collected box (in this case 13 points would be earned). The BRET performed well when compared to other commonly administered risk-taking tasks due to its simplicity and large number of risk categories [71].

The RT-18 [72] is an 18-item binary response questionnaire. It is designed for use in adolescents and young adults, and provides characteristic statements (eg. “I often do things on impulse”) which participants agree or disagree to. The responses are summed (1 = *Yes*; 0 = *No*), with adequate internal reliability in this study (α = .80) and in previous research (α ranging between .74 - .89) [72].

### Statistical analysis

IBM SPSS Statistics 24 for Windows and IBM SPSS Amos 24 for Windows (IMB Corp., Armonk, NY, USA) were used for data analysis. Unless otherwise stated in each individual measure’s scoring guide, participants were excluded if they completed less than 90% of any subscale (6.8%; *n* = 21). Participants (*n* = 3) who did not enter their date of birth or gender were also removed. The excluded individuals did not differ from the rest of the sample. For participants who completed more than 90% of any subscale measure but had not completed the subscale fully, their mean score for the rest of that subscale was used to replace any missing values [73].

**Fig 1:**
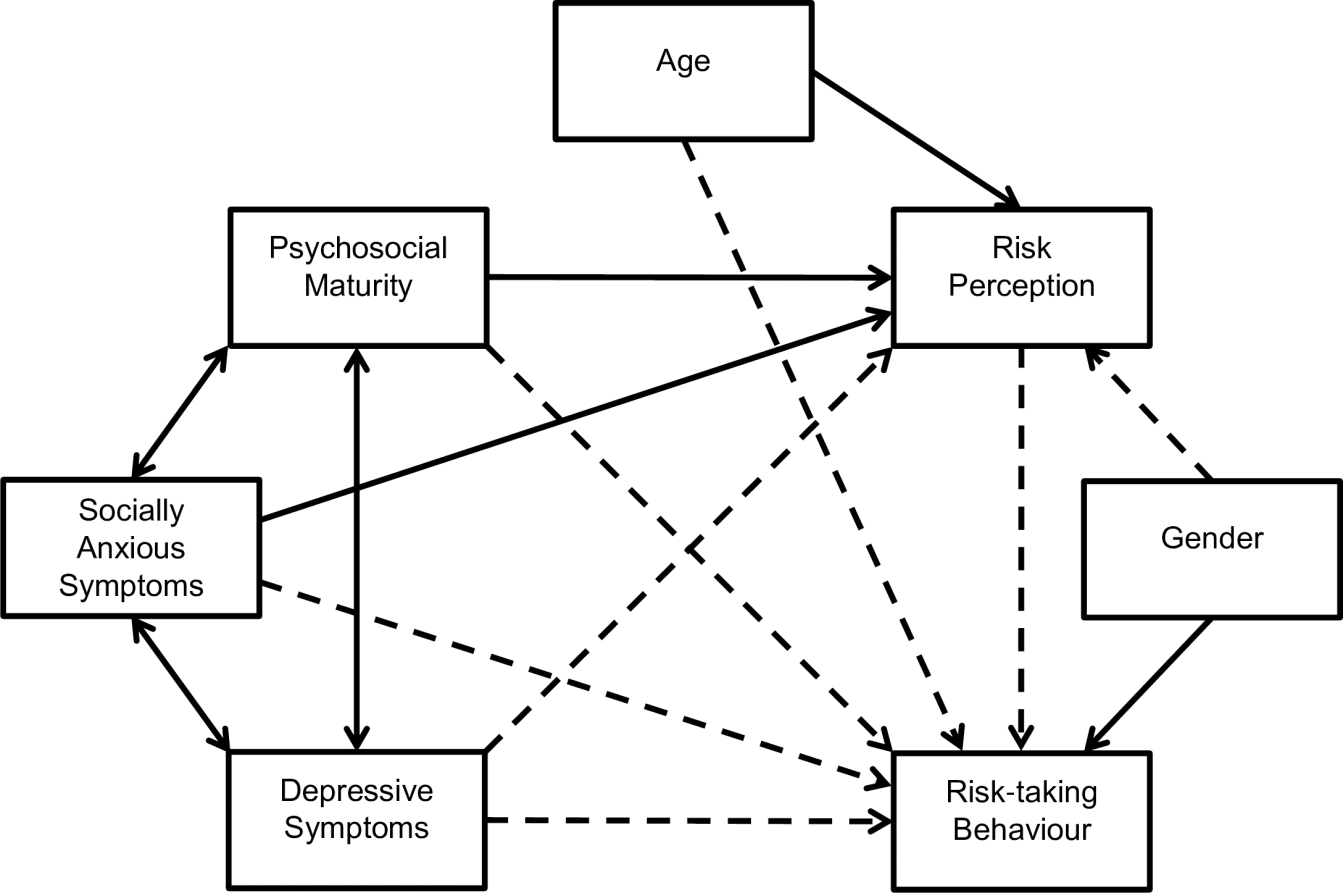
Model displaying the predicted associations between risk-taking behaviour, depressive and socially anxious symptoms, psychosocial maturity, risk perception, age, and gender. Boxes represent observed variables, solid single headed arrows represent positive associations, dashed single headed arrows represent negative associations, and double headed arrows represent correlations. Positive associations for gender indicate an increase in the outcome variable amongst males relative to females.

Path analysis was employed to evaluate the interconnectedness of risk-taking behaviour, depressive and socially anxious symptoms, psychosocial maturity, risk perception, age, and gender. The predicted associations, as outlined in the last paragraph of the introduction, are displayed in Fig 1. Gender was coded with males = 1 and females = 0, so that a positive association indicated that male gender was associated with a greater increase in the outcome variable than female gender. Models were developed using the maximum likelihood method and goodness of fit measures were assessed using guidelines published by Byrne [74] and Ullman [75].

One assumption of structural equation modelling is that the data follows a normal distribution. The Bollen-Stine bootstrap provides a modified method for the χ^2^ goodness-of-fit statistic that is based on a transformation of the data such that the model becomes a perfect fit to the data. Bootstrap samples are drawn with replacement from the transformed data and the distribution of the discrepancy function across all bootstrap samples is taken as an estimate of its distribution under the hypothesis that the model is correct [74,76].

Standardised indirect effects of significant associations were also calculated for predictor variables on risk-taking behaviour through mediator variables. Standardised effects of the predictor variable on the mediator variable were multiplied by the standardised effect of the mediator variable on the outcome variable, risk-taking behaviour. This resulting indirect effect was added to the direct effect the predictor variable had on risk-taking behaviour to give the total effect the predictor variable had upon risk-taking behaviour.

## Results

Descriptive statistics of the measured variables for the whole sample, as well as males and females separately, are presented in Table 1. Distributions for depressive symptoms (*D* = 0.11, *p* < .001), socially anxious symptoms (*D* = .12, *p* <0.001), risk perception (*D* = 0.98, *p* < .001) and age (*D* = 0.17, *p* < .001) were not normal, and therefore median and range for the variables were presented.

**Table 1:** Descriptive statistics of the measured variables.

	Total (*n* = 282)		Female (*n* = 188)		Male (*n* = 94)	
	Median	Min—Max	Median	Min—Max	Median	Min—Max
Age	20.47	16.01–35.85	20.45	16.17–35.85	21.17	16.01–1.70
Risk-Taking Behaviour*	-.04	-1.77–2.30	-.50	-1.89–1.54	.12	-.45–1.70
Risk Perception	2.88	2.00–3.83	2.92	2.00–3.83	2.83	2.04–3.83
Psychosocial Maturity	2.55	.00–5.00	2.58	.21–7.47	2.33	.07–5.00
Depressive Symptoms***	17.00	8.00–39.00	19.00	8.00–39.00	12.50	8.00–33.00
Socially Anxious Symptoms**	35.50	.00–138.00	39.50	.00–138.00	27.00	.00–129.00

Median and range are presented due to non-normality of the results. Variables with an asterix (*) indicate a statistically significant difference between genders

*p < .05

**p < .01

***p < .001.

**Fig 2:**
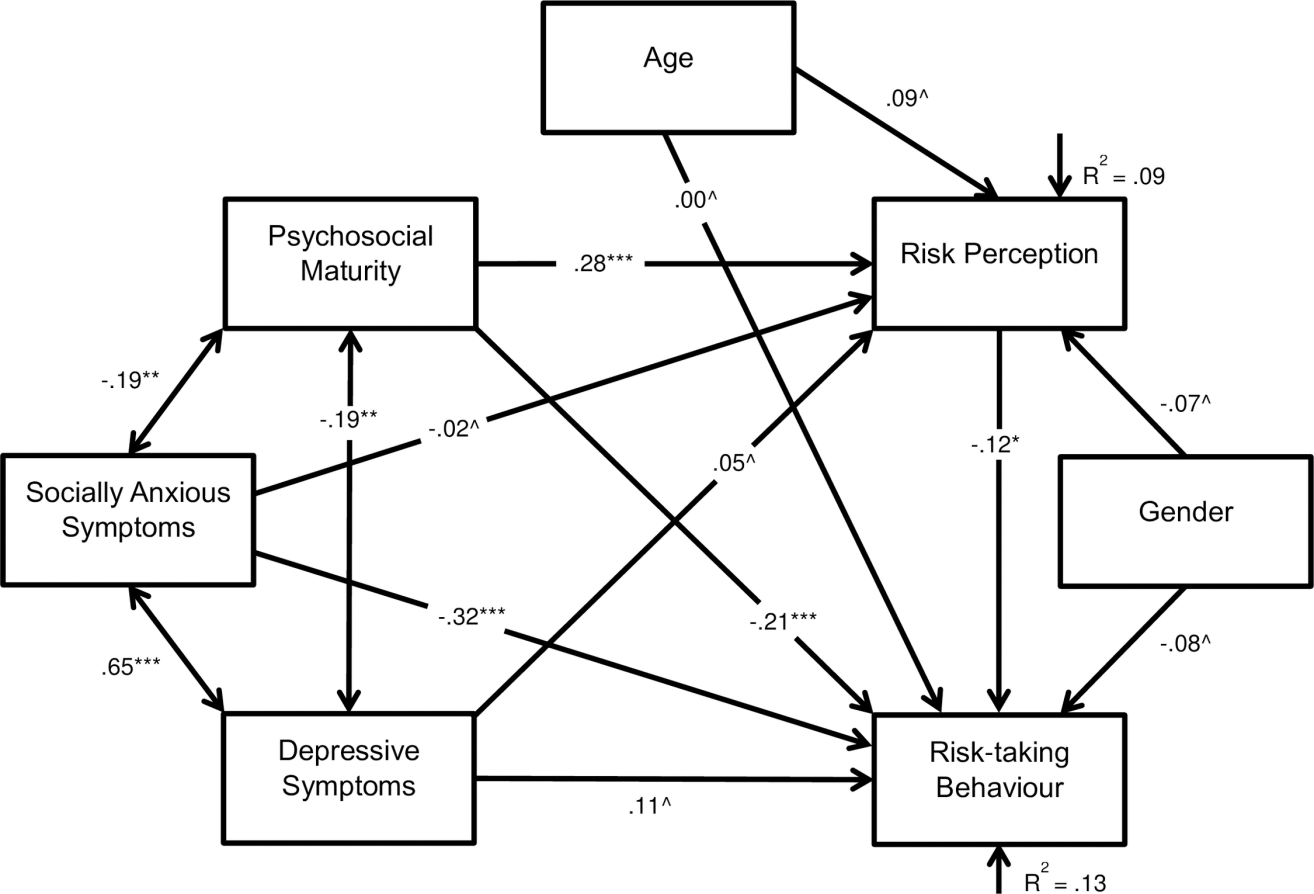
Path model 1 displaying the associations between risk-taking behaviour, depressive and socially anxious symptoms, psychosocial maturity, risk perception, age, and gender. Boxes represent observed variables, long arrows represent regressions, double headed arrows represent correlations, short arrows represent residual error variances, and values represent standardised effect sizes. ^*p* > .05, *p < .05, **p < .01 ***p < .001. R^2^ represents the amount of variance explained by the model.

**Fig 3:**
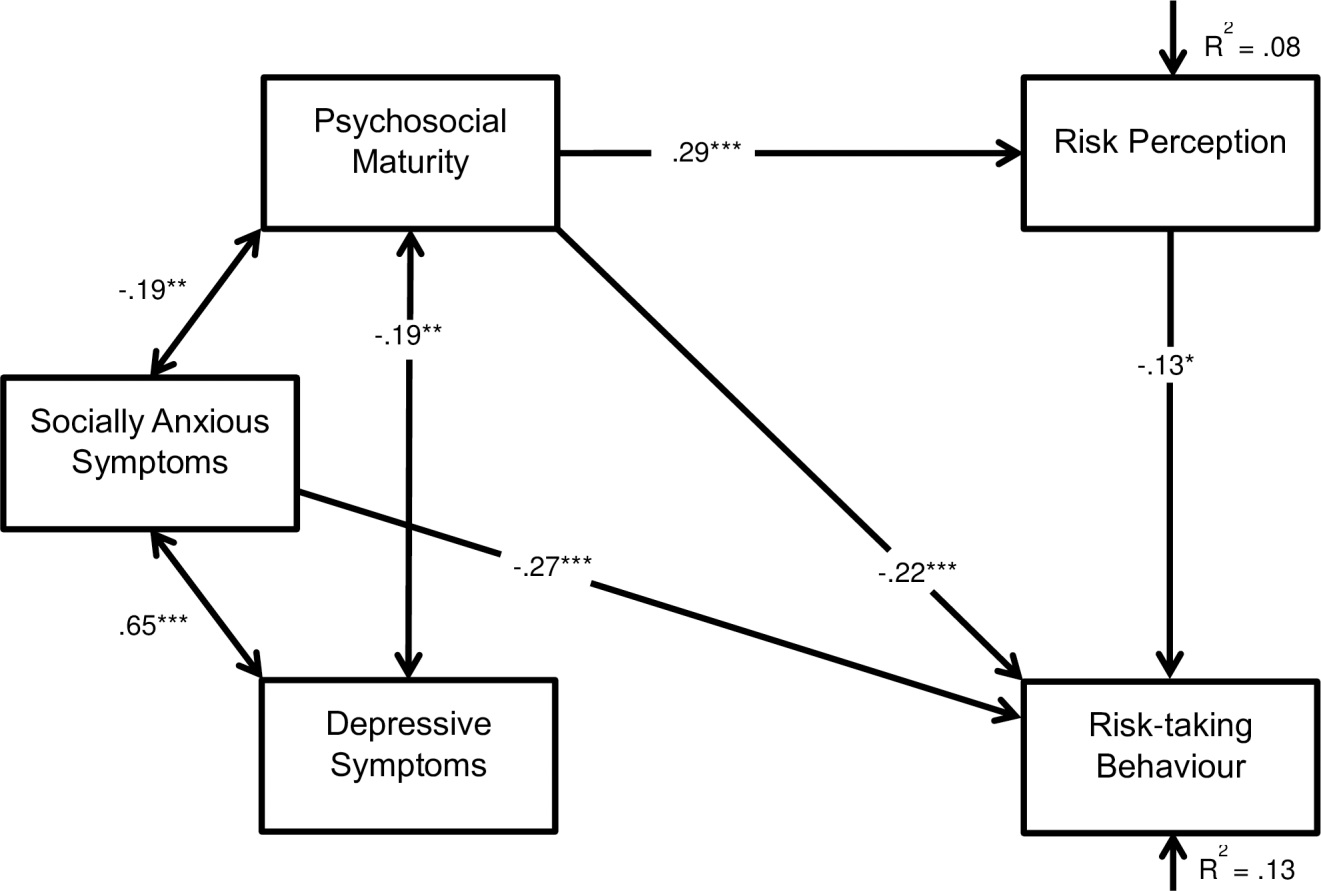
Path model 2 displaying the associations between risk-taking behaviour, depressive and socially anxious symptoms, psychosocial maturity, and risk perception. Boxes represent observed variables, long arrows represent regressions, double headed arrows represent correlations, short arrows represent residual error variances, and values represent standardised effect sizes. *p < .05, **p < .01 ***p < .001. R^2^ represents the amount of variance explained by the model.

The hypothesised model (Fig 1) found socially anxious symptoms to be predictive of a decrease in risk-taking behaviour (*β* = -.32, SE = .00, Z = -4.40, *p* < .001), and the bi-directional association between depressive and socially anxious symptoms was significant (*r* = .65, SE = 17.58, Z = 9.15, *p* < .001). Psychosocial maturity was associated with a decrease in risk-taking behaviour (*β* = -.21, SE = .05, Z = -3.59, *p* < .001) and an increase in risk perception (*β* = .28, SE = .02, Z = 4.80, *p* < .001). Correlations between psychosocial maturity and depressive symptoms (*r* = -.19, SE = .44, Z = -2.13, *p* = .002) and psychosocial maturity and socially anxious symptoms (*r* = -.19, SE = 1.80, Z = -3.14, *p* = .002) were significant. Risk perception was associated with a decrease in risk-taking behaviour (*β* = -.12, SE = .12, Z = -2.10, *p* = .036) (Fig 2). All other associations were insignificant. Model 1 did not fit the data well (Table 2). All insignificant associations were removed to reach model 2 (Fig 3).

**Table 2:** Goodness of fit tests.

		Goodness of fit measure					
Model	Parameters (estimated)	χ^2^(df), *p*	RMSEA (90% CI)	AIC	SRMR	CFI	TLI
1	35 (28)	χ^2^(7) = 28.77, *p* < .001	.11 (.07 - .15)	84.77	.07	.91	.73
2	20 (17)	χ^2^(3) = 2.30, *p* = .513	.00 (.00 - .09)	36.30	.02	1.00	1.00

df = Degrees of freedom, RMSEA = Root Mean Squared Error of Approximation, AIC = Aikaike’s Information Criterion, SRMR = Standardised Room Mean Square Residual, CFI = Bentler’s Comparative Fit Index, and TLI = Tucker-Lewis Index.

Model 2 provided an improved fit to the data (Table 2) with all associations proving significant. The model explained 13% of the variance (*R*^*2*^) in risk-taking behaviour, and 8% of variance in risk perception. Bootstrapping confirmed that the data were a good fit to the model (Bollen-Stine bootstrap *p* = .50). The indirect and total effect psychosocial maturity had upon risk-taking behaviour through mediation with risk perception is displayed in Table 3.

**Table 3:** Standardised total and indirect effects of psychosocial maturity on risk-taking behaviour.

Predictor	Mediator	Indirect effect	S.E.	(95% C.I.)		Total effects
				Lower	Upper	
Psychosocial Maturity	Risk Perception	-0.036	0.02	-0.06	0.00	-0.259

## Discussion

This study evaluated the interconnectedness of risk-taking behaviour, depressive and socially anxious symptoms, psychosocial maturity, risk perception, age, and gender. Path analysis found that symptoms of social anxiety, psychosocial maturity, and risk perception were directly associated with risk-taking behaviour. Depressive symptoms, age, and gender had no direct association with risk-taking behaviour. Psychosocial maturity also had a significant indirect effect on risk-taking behaviour through mediation with risk perception. Correlations between depressive symptoms, socially anxious symptoms, and psychosocial maturity were also significant. The model provided good fit to the data. Psychosocial maturity proved a better predictor for risk-taking behaviour in this study than age.

Adolescence and young adulthood are characterised by identity formation [8], and social interaction plays an important role in this. Young people spend a substantial amount of time with their peers and a growing body of evidence suggests susceptibility to their influence, being it overt or just their mere presence [3,15,16,77]. Besides taking more risks to increase acceptance from peers, meet expectations or achieve status [35–38], it needs to be acknowledged that those who are more anxious in social situations often tend to be more risk avoidant and report to take fewer risks than their less socially anxious counterparts in experimental settings or self-report research. The latter was predicted and observed in the current study; socially anxious symptoms were associated with a decrease in risk-taking behaviour. This potential discrepancy between real life and experimental/self-report settings warrants further investigation, particularly in those most vulnerable to their social environment. Importantly, one’s capacity to resist influences from the social environment continues to develop into young adulthood [3] and efforts to reduce risk-taking behaviour should consider factors in one’s social environment [17]. Depressive and socially anxious symptoms showed a moderate correlation with each other in this study (*r* = .65, *p* < .01) and future research should investigate the extent to which this highly prevalent comorbidity impacts on risk-taking behaviour [78].

Contrary to prediction, depressive symptoms were not directly associated with risk-taking behaviour or risk perception. Whilst evidence of the association between depressive symptoms and health-risk behaviour is building [26,79], findings for other domains of risk-taking are less consistent. Indeed, an association has been found for depressive symptoms with recreational and gambling domains of risk-taking [80], driving performance [81], and theft [82] but not with ethical, health/safety, investing, or social risk-taking [80]. In the current study we conducted a more general assessment of risk-taking behaviour which may have masked a potential association between depressive symptoms and a specific dimension of risk-taking behaviour. The suggestion that depressive symptoms may be most strongly associated with health-related domains of risk-taking places one’s wellbeing in the centre of this relationship and warrants replication of the current study in a clinical sample. Those who are most vulnerable in terms of mental health may be those at highest risk of taking risks, potentially creating a circle in which one enhances the other. The same could perhaps be said for the association between depressive symptoms and risk perception [51], suggesting an important direction for future research.

The lack of a direct effect of depression on risk-taking behaviour does not necessarily mean that there is no effect. An effect between depression and risk-taking behaviour could be indirect, through psychosocial maturity. As an individual matures they will develop greater insight in their own mental health, enabling them to recognise symptoms of mental illness and seek support [39]. Conversely, with increased responsibility and independence, individuals may develop better coping strategies. Therefore, improved insight in one’s mental health, more effective coping mechanisms, and perhaps just a wider range of life experiences, are all factors that may be associated with increased psychosocial maturity, and that may, besides being associated with a reduction in depressive symptoms, also reduce one’s risk-taking behaviour. As the association between depression and psychosocial maturity constituted of a correlation, so was bidirectional rather than an association in a dominant direction, this could not be further investigated in the current model. Future research should aim to explore this indirect association of depression, or potentially other domains of mental health, with risk-taking behaviour. In addition, attempts should be made to expand on the particular aspects of psychosocial maturity that may be associated with mental health. Contrary to prediction, no direct association was observed between socially anxious symptoms and risk perception. This finding may suggest that cognitive appraisal of a risk may not necessarily be influenced by the experienced levels of social anxiety. Fear could cause an individual to appraise a risk as being greater than it is [83–85], triggering avoidance of the behaviour [32,86,87]. Though this could explain why social anxiety is associated with reduced risk-taking behaviour, it does not explain why risk perception remains unchanged. The opportunity to reason about risk without immediate consequences, as is the case in this questionnaire based study, may prevent an individual’s instinctive reactions from taking the overhand. In a developmental period characterised by heightened reactivity to emotions [16,40] this is an important observation, urging young people to stop and think before rushing into action. Adolescents and young adults have a yet immature ability to control their impulses whilst battling their heightened sensitivity to reward and rising levels of sensation seeking [16,41,42]. As this is where questionnaire based research significantly differs from real life behaviour, caution in terms of generalisation of findings is warranted.

Consistent with prediction, psychosocial maturity was associated with a decrease in risk-taking behaviour, both directly and indirectly. Being able to assess a situation and its complexity, and being able to independently make an informed decision—in other words reaching the developmental stage of adulthood—has a positive influence on reducing risk-taking behaviour. Increased psychosocial maturity improves an individual’s maturity of judgement and shifts their perspective from the present to somewhere more future orientated [39]. In this perspective, individuals would appraise the risky behaviour differently as they would be able to better comprehend future consequences [88] and appreciate the full complexity of a situation [44]. This is reflected in the indirect effect of psychosocial maturity on risk-taking behaviour in this study. Being more psychosocially mature may lead to a more mature, and therefore increased, perception of risk, resulting in a reduction of actual risk-taking behaviour.

The current findings indicate that psychosocial maturity presents a better predictor of risk-taking behaviour than age in this study. While psychosocial maturity was directly associated with risk-taking behaviour and risk perception, age showed no association with risk-taking behaviour or risk perception. This is a strong indicator that the length of time a person has lived by itself does not impact on these domains but that instead a multi-dimensional variable, encompassing temperance, perspective, and responsibility as the most important characteristics, has important value as a proxy of development. Psychosocial maturity and age showed a weak correlation in this study (*r* = .13, *p* = .029), thereby demonstrating their independence as domains of temporality and development. Importantly, these findings inform popular models of adolescent development. They support models such as the dual systems model which demonstrate the importance of the consideration of maturity in risk-taking behaviour but also decision-making more generally [40]. Individuals develop at different rates, biologically and psychosocially, and this should be taken into account when considering behaviour.

Despite gender differences in risk-taking behaviour being observed, with males taking significantly more risks than females, gender did not prove a significant domain in the current study. This may be because of its relevance to psychosocial maturity. Women are generally more psychosocially mature than their male counterparts [89], suggesting that the impact of gender may have already been accounted for in this domain. Further research should aim to disentangle potential gender differences from psychosocial maturity.

A strength of this study is the multi-dimensional approach to measuring risk-taking behaviour, evaluating the interconnectedness of domains (mental health and psychosocial maturity) that have kept relatively under the radar in the investigation of their associations with risk-taking behaviour and risk perception. Despite this, the study is limited in that the BRET is not a repeated measure. Further, a financial incentive could not be provided for the study participants completing the BRET, limiting the real-life aspect of risk-taking behaviour. Development is an ongoing process and the current findings should be considered within their limitation of cross-sectional research. Longitudinal follow up of individuals developing through adolescence and young adulthood would be the definitive way of reaching full understanding of the causality of the interactions that may underlie these complex behaviours. Furthermore, the current study limited itself to the evaluation of the interconnectedness of a selected number of domains. Whilst the specific focus on these domains constituted the aim of the current study, the importance of other factors, such as one’s genetic makeup and personality, socioemotional factors (e.g. the influence of peers), psychological stress, and hormonal balance should not be forgotten. Moreover, while depression and social anxiety are classed as common mental disorders [21,90], this does not automatically mean that the observed associations would be equally applicable to other mental disorders. Research investigating generalisation to other anxiety disorders and mental health more broadly is warranted.

Taken together, the findings of the current study indicate that mental health impacts upon risk-taking behaviour and that consideration should be given to psychosocial maturity in attempts to reduce adolescent and young adult risk-taking behaviour. Consequently, it could be recommended that screening for mental health should be systematically undertaken during consultations whilst risk-taking behaviour should be monitored when treating symptoms of mental illness [79]. In addition, this would mean targeting those who display symptoms of mental illness and are considered less mature, rather than those of a particular age, in attempts to reduce adolescent and young adult risk-taking behaviour.
